# Removal of Pathogens from Domestic Wastewater Using Small-Scale Gradual Hydroponics Planted with *Duranta erecta*, Addis Ababa, Ethiopia

**DOI:** 10.1155/2022/3182996

**Published:** 2022-01-05

**Authors:** Solomon Tibebu, Abebe Worku, Kenatu Angassa

**Affiliations:** ^1^Department of Environmental Engineering, College of Biological and Chemical Engineering, Addis Ababa Science and Technology University, Addis Ababa 16417, Ethiopia; ^2^Sustainable Energy Center of Excellence, Addis Ababa Science and Technology University, Addis Ababa 16417, Ethiopia; ^3^Bioprocess and Biotechnology Center of Excellence, Addis Ababa Science and Technology University, Addis Ababa 16417, Ethiopia

## Abstract

This study aimed to evaluate the treatment potential of gradual hydroponics planted with *Duranta erecta* in the removal of pathogens from domestic wastewater. Two experimental and control units were configured in series. Each unit contains three bioreactors and was arranged in a cascaded configuration. The two experimental units used both plant and media, but the two control units used only media to treat the wastewater. Gravel and polyester sponge were used as media. Experimental unit 1 and control unit 1 used gravel as media; however, experimental unit 2 and control unit 2 used polyester sponges as media. The experiment was operated at hydraulic retention times of 1, 3, 5, and 7 days in a continuous mode. The performance of the hydroponic system was evaluated by characterizing the influent and effluent quality using standard methods. At optimum hydraulic retention time (7 days), the average removal of experimental units 1 and 2 was 98.7% and 89.8% for heterotrophic bacteria, 96.2% and 86.8% for total coliform, and 92.9% and 84.0% for fecal coliform, respectively. Analysis of variance showed that there was a significant difference (*P* < 0.05) between the two experimental and control units in removing pathogens, but no significant difference (*P* > 0.05) was observed between the two experimental units and between the two control units. Heterotrophic bacteria and coliforms were satisfactorily removed from domestic wastewater via a gradual hydroponic system. Hence, the hydroponic treatment system planted with *Duranta erecta* has a promising potential in the removal of pathogens from domestic wastewater in developing countries including Ethiopia.

## 1. Introduction

Domestic wastewater disposal has become a serious problem in urban areas of developing countries [1]. A large portion of wastewater is discharged directly into nearby surface water bodies or informal drainage channels, often without or with little treatment, especially in developing countries including Ethiopia [2, 3]. Globally, surface water quality is rapidly becoming a scarce resource; on the other hand, surface water quality such as river water quality is anthropologically being affected [4–8]. In Addis Ababa, rivers such as *Fanta* and *Akaki* rivers have been impaired for many years due to anthropological activities. Because of an increasing population, urban farming, industrial expansion, and lack of sufficient sewage treatment, the city of Addis Ababa is suffering from serious surface water pollution [9–12].

Wastewater and refuse disposal is often the most intractable sanitation problem in places of detention [13]. The wastewater generated from the *Kilinto* Prison camp, which is one of the federal prisons located on the outskirts of Addis Ababa, is being directly discharged into a small river called the *Fanta* River. At the time of this study, 264 m^3^/d of untreated effluent is being discharged to the *Fanta* River, which is located upstream of Big *Akaki* River. Big *Akaki* River is one of the most polluted rivers in Addis Ababa [12, 14–16]. There are many reasons for the pollution of this river, among which is the contamination of its tributaries including the *Fanta* River. The *Fanta* River is contaminated by untreated wastewater discharges and runoff from residential settlements in its upstream catchment. Thus, the major sources of pollution in the *Fanta* River are residential districts and predominantly public institutional facilities that impose environmental burdens. In vegetables, grown by the river water in the studied areas, fecal coliforms and a high colony of E. coli were identified. This might cause an increase in nonspecific diarrhea for consumers, exceeding 104 per 100 ml [17]. The farmers are often exposed to protozoa, which raise the risk of *amoebiasis*; for example, direct contact with irrigation water polluted with wastewater triggers skin irritations such as dermatitis [18, 19]. Indicator organisms (coliforms) were used to detect the presence of pathogens [20–22].

Nature-based wastewater treatment and reuse concepts are growing as a naturally focused low-cost wastewater treatment and reuse technology, where wastewater is treated biologically including constructed wetland and hydroponic technology [23]. Determining the source and type of wastewater is very important to determine its characteristics, which in turn helps to select technology and treat the given wastewater properly [24].

Hydroponics is one of the recent wastewater treatment technologies that uses a symbiotic relationship between plants and microorganisms to treat wastewater [25, 26]. It is the growing of plants in a liquid nutrient solution or wastewater with or without the use of media [27–29]. The plants diffuse oxygen through the rhizosphere and provide surface area for the attachment of microorganisms through their roots [30]. The microbes attached to the plant root will fix nutrients and degrade organic matter so that the plant can absorb them [31]. Hydroponic wastewater treatment technology has the potential to remove microorganisms from wastewater satisfactorily [32]. An aggregate hydroponic system was employed to enhance the growth of microorganisms by providing more surface area for the attachment in addition to the surface area provided by the plant root [30]. Sedimentation, filtration, predation by other organisms, natural die-off, and the release of root exudates are some of the main mechanisms for bacteria removal in aggregate hydroponics [32–34]. Hydroponics has many advantages over conventional and other nonconventional wastewater treatment technologies. Most conventional wastewater treatment technologies have high investment costs. They also have high operational and maintenance costs, and most nonconventional wastewater treatment technologies require large areas [27]. Hydroponic wastewater treatment technologies have relatively low operational and maintenance costs; therefore, they are preferable for developing countries including Ethiopia [35].

Ornamental plants are very important in environmental management and beautification [36]. There are many ornamental plants in Ethiopia such as *Nerium oleander, Pinus patula, Euphorbia cotinifolia, Cordyline terminalis,* and *Duranta erecta* [37]. In this study, a hydroponic system planted with *Duranta erecta* was designed, arranged, and operated to assess the removal potential of pathogens from domestic wastewater*. Duranta erecta* is the most frequently used ornamental plant in Ethiopia. It is a fast-growing and multistemmed shrub plant. It grows in tropical and warm subtropical regions and suits for gardens. In areas that have infertile natural conditions and drought, they tend to grow up to 1 m, and in watered and fertilized areas, they grow up to 3 m. *Duranta erecta* has a moderate growth rate, usually 0.5 m/year for the first few years. They can live for at least 15 years [38]. Hence, the plant is available and suitable to grow hydroponically for assessing its wastewater treatment potential.

There are few studies regarding the use of gradual hydroponics for wastewater treatment [39]. It is also noted that no other study has been carried out regarding the use of *Duranta erecta* in hydroponic wastewater treatment technology for the removal of pathogens. Therefore, this study aimed to evaluate the treatment potential of gradual hydroponics planted with *Duranta erecta* in the removal of pathogens from domestic wastewater. The technology needs further researches to be utilized as an alternative decentralized wastewater treatment mechanism for developing countries including Ethiopia.

## 2. Materials and Methods

### 2.1. Description of the Study Area

**Figure 1:**
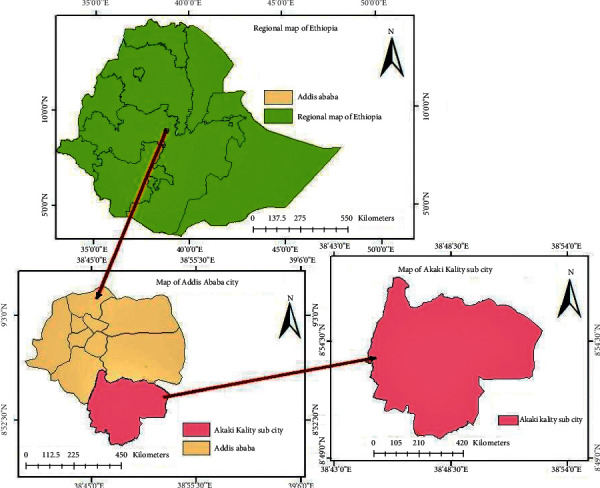
Experimental site.

The experimental site is located in Akaki Kality sub-city, Addis Ababa, Ethiopia, as shown in Figure 1. The city is located at ″9^o^ 02′01.76″ N and 38^o^45′18.33″ E″ and has an altitude of 2300 m. It has an average temperature, rainfall, and relative humidity of 15.9°C, 1089 mm, and 60.7%, respectively.

### 2.2. Experimental Design and Operation of the Hydroponic System

**Figure 2:**
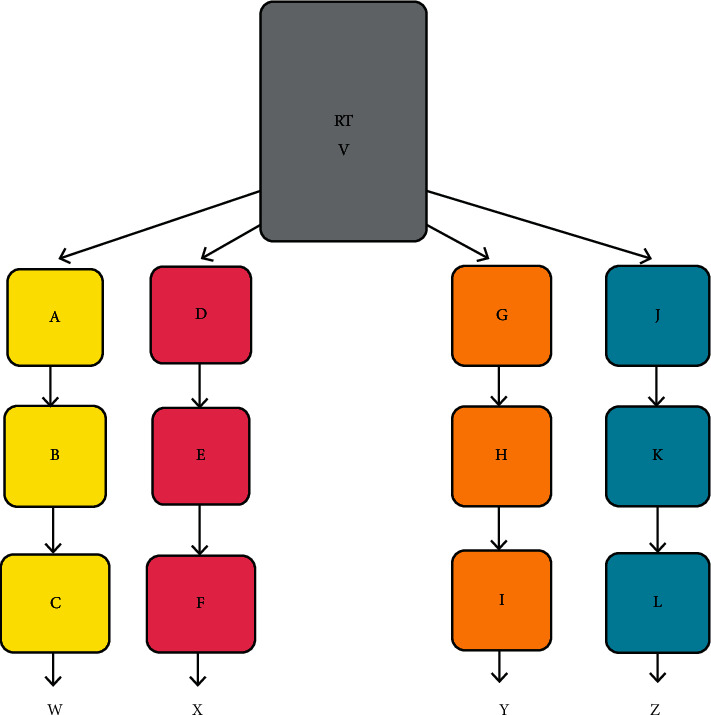
Schematic diagram of the experimental setup.

Two experimental and two control units were configured in series as shown in Figure 2. Each experimental and control unit contained three bioreactors. Each of these units was employed to treat 60 L wastewater at a given hydraulic retention time (HRT).

Where A, B, and C denote bioreactors in experimental unit 1 (E-1); D, E, and F denote bioreactors in control unit 1 (C-1); G, H, and I denote bioreactors in experimental unit 2 (E-2); J, K, and L denote bioreactors in control unit 2 (C-2); RT denotes the reservoir tank; and V, W, X, Y, and Z are sampling ports.

In E-1 and C-1, gravel was used as media, but in E-2 and C-2, a polyester sponge was used as media. Healthy and young plants were collected from Afincho Ber area in Addis Ababa near the experimentation site. The root of the plants was carefully washed to remove the adhered soil material. The plant's root had been trimmed to 11 cm, and five young plants were planted per reactor in E-1 and E-2. On each reactor lid, a forestry tube was used to suspend the plant along with the media. 387.28 g of sharp gravel, which has a diameter of 1 cm, is placed in each forestry tubes on both E-1 and C-1. 29.28 g of a polyester sponge is placed in each forestry tubes on both E-2 and C-2. The porosity of gravel and polyester sponge is 0.44 and 0.68, respectively. The experimental units contain both media and plants, but the control units only contain media.

After planting, acclimatization was conducted for about three months to adapt to the stress of the plants due to high organic loadings [40]. First, tap water was used and then wastewater concentration was increased to 5%, 15%, 20%, 30%, 50%, 80%, and finally, 100% was achieved. Pollutant load increment is conducted every eight days of interval. The untreated wastewater was collected from the direct discharge point of Kilinto Federal Prison camp, transported, and fed to the reservoir tank. The same raw wastewater sample sourced from the reservoir tank was fed to all treatment and control units in a continuous mode. The experiment was operated at four HRTs, i.e., 1, 3, 5, and 7 days [41]. A 6 mm tap valve was used to control the flow of the wastewater from the reservoir tank (RT) and from each experimental and control bioreactor.

### 2.3. Wastewater Sampling and Analysis

Four composite samples (one sample per month) were taken from the direct discharge point of *Kilinto* Prison camp to characterize raw wastewater. Grab sampling was used to collect samples from the influents and effluents of the hydroponic treatment system to evaluate the performance of the treatment system in terms of the removal of pathogens. For each HRT (i.e., 1, 3, 5, and 7 days), one sample was taken from influent and four samples were taken from effluents (i.e., E-1, E-2, C-1, and C-2). A triplicate water sample analysis was conducted according to standard methods. Totally, 20 triplicate sample analyses were conducted according to [42] standard methods for the examination of water and wastewater to evaluate the performance of the experimental and control units. Spread Plate Method 9215 was used to analyze heterotrophic bacteria, and Membrane Filter Method 9222 was used to analyze both total and fecal coliforms.

### 2.4. Data Analysis

The generated data were analyzed by Microsoft Excel 2013. One-way analysis of variance (ANOVA) was used to compare the treatment performance of hydroponic units for the heterotrophic bacteria, total coliform, and fecal coliform removal with a 95% confidence interval. A linear correlation was also observed to determine the correlation between HRT and removal efficiency. The removal efficiency was calculated based on the following equation:

(1) $$\begin{array}{c} \eta =\left(\frac{C1-C2}{C1}\right)*100, \end{array}$$

where *ƞ* is removal efficiency, *c*_1_ is influent concentration, and *c*_2_ is effluent concentration.

## 3. Results and Discussion

### 3.1. Characteristics of the Wastewater

The *Kilinto* Prison camp wastewater was characterized from August 2019 to November 2019. The average (Avg) heterotrophic bacteria (HB), total coliform (TC), and fecal coliform (FC) counts (log 10 units) in the effluent ranged from 7.94 to 8.63 CFU/100 ml, 5.70 to 5.89 CFU/100 ml, and 4.37 to 5.14 CFU/100 ml, respectively, as shown in Table 1.

It can be noted that the colony of bacteria is increasing from August to November as can be seen in Table 1. This might be due to the change in climatic conditions and runoff dilution of the effluent in the sewerage system through leaky joints. These factors cause a decrease in bacterial colonies in the two Ethiopian wet seasons (August and September). However, in October and November, the rainfall intensity decreased. This in turn decreased the dilution and increased the pollutant load, which leads to the increment of the bacterial population.

The presence of high coliforms in the effluent indicates the presence of pathogenic organisms that can cause health effects on humans [43]. Dysentery, cholera, typhoid, fever, and diarrhea are some of the potential health risks, which might be caused by these pathogenic microorganisms in untreated or poorly treated domestic wastewater including the case of this study [44–46]. Therefore, treating such wastewater types containing pathogenic microorganisms before discharge into the environment is mandatory.

### 3.2. Performance of Gradual Hydroponics

The mean influent value of HB, TC, and FC counts (log 10 units) during the experiment varied between 8.76 ± 0.020 and 8.95 ± 0.016 CFU/100 ml, 5.54 ± 0.005 and 5.86 ± 0.002 CFU/100 ml, and 4.55 ± 0.020 and 4.96 ± 0.008 CFU/100 ml, respectively. At 1, 3, 5, and 7 days of HRT, the mean effluent concentration of HB, TC, and FC counts (log 10 units) during the experiment varied between 7.06 ± 0.098 and 8.34 ± 0.004 CFU/100 ml, 4.43 ± 0.128 and 5.17 ± 0.021 CFU/100 ml, and 3.80 ± 0.125 and 4.25 ± 0.047 CFU/100 ml, respectively, for E-1. For E-2, the mean effluent concentration of HB, TC, and FC counts (log 10 units) for the same HRT varied between 8.43 ± 0.005 and 7.96 ± 0.015 CFU/100 ml, 5.25 ± 0.018 and 4.99 ± 0.019 CFU/100 ml, and 4.34 ± 0.032 and 4.17 ± 0.046 CFU/100 ml, respectively. Similarly, At 1, 3, 5, and 7 days of HRT, the mean effluent concentration of HB, TC, and FC counts (log 10 units) during the experiment varied between 8.63 ± 0.003 and 8.38 ± 0.007 CFU/100 ml, 5.46 ± 0.011 and 5.55 ± 0.008 CFU/100 ml, and 4.47 ± 0.031 and 4.68 ± 0.013 CFU/100 ml, respectively, for C-1. For C-2, the mean effluent concentration of HB, TC, and FC counts (log 10 units) for the same HRT varied between 8.68 ± 0.003 and 8.56 ± 0.004 CFU/100 ml, 5.49 ± 0.012 and 5.68 ± 0.007 CFU/100 ml, and 4.50 ± 0.030 and 4.79 ± 0.011 CFU/100 ml, respectively. The mean concentration of influent and effluent from each experimental unit and control unit is described in Table 2.

A reduction in the concentration of HB, TC, and FC in effluents was observed than that of influents in all experimental and control units. E-1 showed higher performance in removing HB, TC, and FC followed by E-2, C-1, and C-2, respectively, in all applied HRTs. The removal of all three parameters in E-1 and E-2 might be caused by sedimentation, predation by other organisms, and natural die-off [32]. The release of root exudates might also play a vital role in the removal of microorganisms by being toxic to pathogenic microbes [33]. Root exudates are released when the root cell senses the presence of pathogenic microorganisms. The change in the physicochemical environment due to the release of root exudates might also contribute to the removal of pathogenic microorganisms [47]. The removal of all three parameters in C-1 and C-2 might be caused by sedimentation, adsorption, predation by other organisms, and natural die-off. C-1 showed better performance in removing the above three parameters than that of C-2. This might be because of the better provision of adsorption site for the particles, which in turn leads to a better provision of attachment site for the microbes by gravel than the polyester sponge. When particles are removed from the given wastewater, microbes will also be removed because microbes are attached to the particle. Because of this reason, the removal of bacteria in C-1 was enhanced by sedimentation and adsorption of particles. Some studies such as [48] stated that the main removal mechanisms of coliforms are predation by other organisms and physicochemical conditions. However, in this study, experimental units, which were planted with Duranta erecta, showed better bacteria removal efficiency than unplanted control units. This indicates that the main bacteria removal mechanism is the presence of the plants and their species. Some studies such as [49] support the fact that the presence of plants can play a vital role in bacteria removal.  Table 3 shows the variance between different groups.

**Figure 3:**
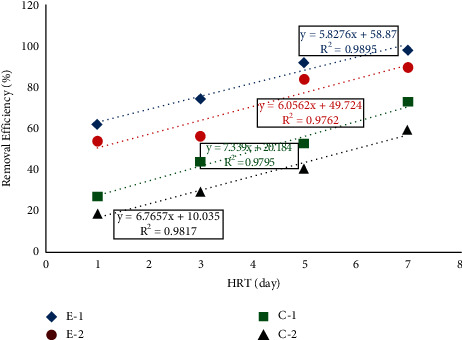
Regression analysis and removal efficiency of HB.

**Figure 4:**
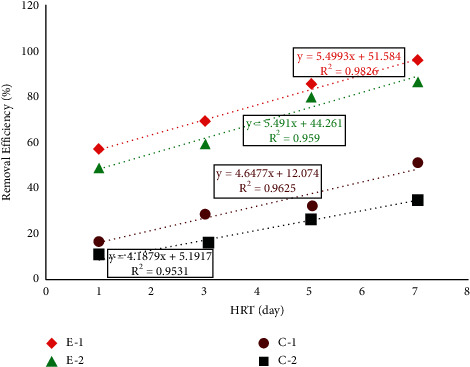
Regression analysis and removal efficiency of TC.

**Figure 5:**
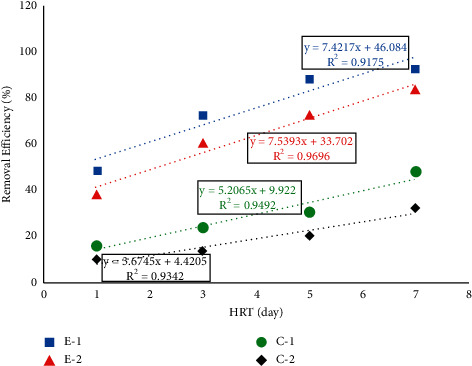
Regression analysis and removal efficiency of FC.

According to Table 3, no significant difference (*P* > 0.05) was observed between the two experimental units (E-1 and E-2) and between the two control units (C-1 and C-2). However, a significant difference (*P* < 0.05) was observed between the two experimental and control units (E-1 and C-1 and E-2 and C-2). The removal performance of the hydroponics in removing HB, TC, and FC ranged between 62.4 and 98.7%, 57.14 and 96.21%, 48.7 and 92.9%, respectively, for E-1 and 54.1 and 89.8%, 49.14 and 86.75%, 37.99 and 84.01%, respectively, for E-2 at HRT of 1, 3, 5, and 7 days. Similarly, the HB, TC, and FC removal performance of hydroponics ranged between 27.2 and 73.3%, 17.14 and 51.35%, and 15.73 and 48.28%, respectively, for C-1 and 18.3 and 59.6%, 11.42 and 35.13%, and 10.14 and 32.42%, respectively, for C-2 at HRT of 1, 3, 5, and 7 days as shown in Figures 3, 4, and 5. The above results show that gradual hydroponics planted with *Duranta erecta* showed high performance in removing HB, TC, and FC. Therefore, gradual hydroponics can be developed into a technology that can be used as alternative decentralized wastewater treatment mechanisms, especially for developing countries including Ethiopia.

The TC removal performance of this study was compared with previous studies. Yeboah and Allotey [50] reported a removal efficiency of 44.4% in treating industrial wastewater using horizontal flow hydroponics, which is less than the removal efficiency recorded in both E-1 and E-2 of this study. The removal efficiency of 60–88.7% was reported by Ottoson and Norström [32] in the treatment of municipal wastewater using horizontal flow hydroponics, which is less than the removal efficiency recorded in E-1, but greater than the removal efficiency that was recorded in E-2 of this study. Similarly, De Anda and López-López [51] reported a removal efficiency of 90.9% in the treatment of domestic wastewater using horizontal flow hydroponics, which is less than the removal efficiency recorded in E-1, but greater than the removal efficiency recorded in E-2 of this study. A better removal efficiency (99%) was reported by Sklarz and Gross [52] and Tunçsiper and Ayaz [53] than both E-1 and E-2 in removing TC from grey and surface water using vertical gravel bed wetland planted with Juncus alpigenus and iris, respectively.

The FC removal efficiency of this study was also compared with previous studies. The removal efficiency of 90% was reported by De Anda and López-López [51] in the treatment of domestic wastewater using horizontal flow hydroponics, which is less than the removal efficiency recorded in E-1, but greater than the removal efficiency recorded in E-2 of this study. Ndulini and Sithole [54] reported a removal efficiency of 92% in the treatment of domestic wastewater using gravel bed hydroponics, which aggresses with the removal efficiency recorded in E-1, but greater than the removal efficiency recorded in E-2 of this study. Generally, this study showed that a better TC and FC removal efficiency was achieved, especially in E-1 compared with the above previous studies. A better removal efficiency (99%) was reported by Abidi and Kallali [55] than E-1 in removing FC from domestic wastewater using vertical gravel bed wetland planted with *Phragmites australis*. Similar removal efficiency (84%) was reported by Sehar and Naeem [56] in removing FC from domestic wastewater using vertical gravel bed wetland planted with *Bulbophyllum reptans* and *Trianthema portulacastrum.*

**Figure 6:**
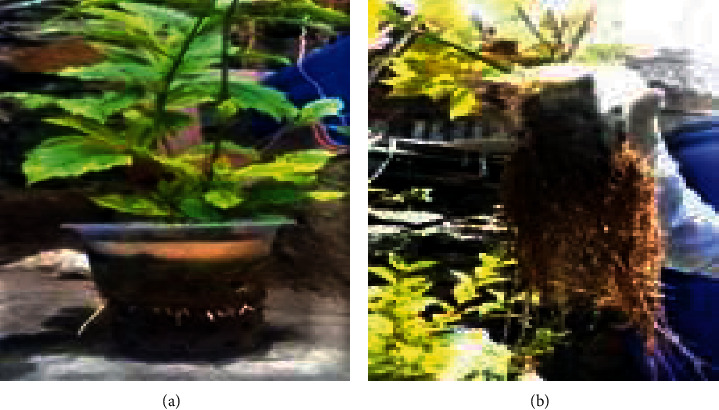
The growth of the plant (*Duranta erecta*) on the forestry tube in vertical hydroponics at two weeks of stages of acclimatization (a) and at the end of the experiment (five months after the plants were planted) (b).

In all three parameters, E-1 showed a better bacteria removal efficiency than E-2. This might be due to the better growth of root biomass in E-1 than that in E-2. The growth of the root biomass enhances the secretion of root exudates. E-1 and E-2 also showed a better bacteria removal efficiency than their corresponding control units.  Figure 6 shows a sample of the plant's roots growing on a forestry tube, which is placed on a reactor in a hydroponic system.

A strong linear correlation was observed in regression analysis between the applied HRTs and removal efficiency of the treatment system in removing HB, TC, and FC as shown in Figures 3, 4, and 5. In the removal of HB, the regression analysis showed that *R*^2^ = 0.989, 0.976, 0.979, and 0.981 for E-1, E-2, C-1, and C-2, respectively. In the removal of TC, the regression analysis showed that *R*^2^ = 0.982, 0.959, 0.962, and 0.953 for E-1, E-2, C-1, and C-2, respectively. In the removal of FC regression, analysis showed that *R*^2^ = 0.917, 0.969, 0.949, and 0.934, respectively. From this, we can understand that the removal of bacteria depends on HRT, which in turn depends on HLR.

## 4. Conclusion

The effluent from the *Kilinto* Prison camp was treated using gradual hydroponics operated at HRT of 1, 3, 5, and 7 days. The optimum HRT in the removal of pathogens was achieved at an HRT of 7 days. The findings showed that at optimum HRT (7 days), a removal efficiency of 98.7 and 89.8% was achieved for HB, that of 96.2 and 86.8% was achieved for TC, and that of 92.9 and 84.0% was achieved for FC using E-1 and E-2, respectively. The results obtained in this study show that the experimental units of gradual hydroponics planted with *Duranta erecta* significantly removed HB, TC, and FC from domestic wastewater. Therefore, scaling up of this pilot gradual hydroponics has a great advantage for developing countries such as Ethiopia.

## Figures and Tables

**Table 1:** Bacteriological characteristics of Kilinto Prison camp wastewater.

Parameter	August	September	October	November
	Avg ± SD^*∗*^	Avg ± SD	Avg ± SD	Avg ± SD
HB (CFU/100 ml)	7.94 ± 0.057	8.63 ± 0.156	8.40 ± 0.019	8.35 ± 0.014
TC (CFU/100 ml)	5.72 ± 0.004	5.70 ± 0.017	5.89 ± 0.004	5.73 ± 0.007
FC (CFU/100 ml)	4.37 ± 0.102	5.14 ± 0.079	4.39 ± 0.081	4.46 ± 0.099

^*∗*^Standard deviation.

**Table 2:** Mean influent and effluent concentration of HB, TC, and FC at different HRTs.

Parameters	HRT (day)	Influent	Effluent				Ethiopian standard
			E-1 Avg ± SD*∗*	E-2 Avg ± SD	C-1 Avg ± SD	C-2 Avg ± SD	
HB (CFU/100 ml)	1	8.76 ± 0.020	8.34 ± 0.004	8.43 ± 0.005	8.63 ± 0.003	8.68 ± 0.003	—
TC (CFU/100 ml)		5.54 ± 0.005	5.17 ± 0.021	5.25 ± 0.018	5.46 ± 0.011	5.49 ± 0.012	2.6
FC (CFU/100 ml)		4.55 ± 0.020	4.25 ± 0.047	4.34 ± 0.032	4.47 ± 0.031	4.50 ± 0.030	1
HB (CFU/100 ml)	3	8.81 ± 0.019	8.21 ± 0.009	8.45 ± 0.006	8.55 ± 0.004	8.66 ± 0.002	—
TC (CFU/100 ml)		5.62 ± 0.005	5.10 ± 0.031	5.23 ± 0.015	5.47 ± 0.010	5.55 ± 0.007	2.6
FC (CFU/100 ml)		4.71 ± 0.017	4.15 ± 0.039	4.31 ± 0.040	4.59 ± 0.019	4.65 ± 0.015	1
HB (CFU/100 ml)	5	8.82 ± 0.010	8.50 ± 0.020	8.02 ± 0.014	8.49 ± 0.004	8.60 ± 0.003	—
TC (CFU/100 ml)		5.71 ± 0.004	4.87 ± 0.054	5.02 ± 0.031	5.54 ± 0.009	5.57 ± 0.004	2.6
FC (CFU/100 ml)		4.77 ± 0.009	3.81 ± 0.110	4.19 ± 0.049	4.61 ± 0.018	4.67 ± 0.012	1
HB (CFU/100 ml)	7	8.95 ± 0.016	7.06 ± 0.098	7.96 ± 0.015	8.38 ± 0.007	8.56 ± 0.004	—
TC (CFU/100 ml)		5.86 ± 0.002	4.43 ± 0.128	4.99 ± 0.019	5.55 ± 0.008	5.68 ± 0.007	2.6
FC (CFU/100 ml)		4.96 ± 0.008	3.80 ± 0.125	4.17 ± 0.046	4.68 ± 0.013	4.79 ± 0.011	1

*∗*Standard deviation.

**Table 3:** Analysis of variance (ANOVA) between different groups.

No.	Parameter	Group	*P* value	Significance (*P* < 0.05)	F-test
**1**	HB	E-1 and C-1	0.0111423	Significant	13.079828
		E-2 and C-2	0.006203	Significant	16.989127
		E-1 and E-2	0.324526	Not significant	1.1512903
		C-1 and C-2	0.375934	Not significant	0.9141555

**2**	TC	E-1 and C-1	0.000403	Significant	49.884600
		E-2 and C-2	0.0010449	Significant	34.915459
		E-1 and E-2	0.265333	Not significant	1.5086709
		C-1 and C-2	0.19356	Not significant	2.1429313

**3**	FC	E-1 and C-1	0.000976	Significant	35.838472
		E-2 and C-2	0.004977	Significant	18.670581
		E-1 and E-2	0.086845	Not significant	4.1818763
		C-1 and C-2	0.407966	Not significant	0.7912619

## Data Availability

All data are available in this study.
